# Adhesion and Transparency Enhancement between Flexible Polyimide-PDMS Copolymerized Film and Copper Foil for LED Transparent Screen

**DOI:** 10.3390/polym16111591

**Published:** 2024-06-04

**Authors:** Xinming Wang, Yuting Zhao, Heming Li, Weiguo Gao, Yan Liu, Anning Sun, Ke Ma, Zhizhi Hu, Yongqi Wang

**Affiliations:** 1 School of Chemical Engineering, University of Science and Technology Liaoning, Anshan 114051, China; wangxm0410@163.com (X.W.); mkqiuzhi@163.com (Y.Z.); lhm900308@163.com (H.L.); gao990322@163.com (W.G.); 18523501103@163.com (Y.L.); sun15964705350@163.com (A.S.); 2 Oxiranchem Holding Group Co., Ltd., Liaoyang 111003, China; 3 School of Information and Engineering, Liaoning Agricultural Technical College, Yingkou 115009, China

**Keywords:** peel adhesion, transparency, polyimide, PDMS, trifluoromethyl

## Abstract

With the increasing demand for innovative electronic products, LED transparent screens are gradually entering the public eye. Polyimide (PI) materials combine high temperature resistance and high transparency, which can be used to prepare flexible copper-clad laminate substrates. The physical and chemical properties of PI materials differ from copper, such as their thermal expansion coefficients (CTEs), surface energy, etc. These differences affect the formation and stability of the interface between copper and PI films, resulting in a short life for LED transparent screens. To enhance PI-copper interfacial adhesion, aminopropyl-terminated polydimethylsiloxane (PDMS) can be used to increase the adhesive ability. Two diamine monomers with a trifluoromethyl structure and a sulfone group structure were selected in this research. Bisphenol type A diether dianhydride is a dianhydride monomer. All three of the above monomers have non−coplanar structures and flexible structural units. The adhesion and optical properties can be improved between the interface of the synthesized PI films and copper foil. PI films containing PDMS 0, 1, 3, and 5 wt% were analyzed using UV spectroscopy. The transmittance of the PI-1/3%, PI-1/5%, PI-2/3%, and PI-2/5% films were all more than 80% at 450 nm. Meanwhile, the T_d 5%_ and T_d 10%_ heat loss and Tg temperatures decreased gradually with the increase in PDMS. The peel adhesion of PI-copper foil was measured using a 180° peel assay. The effect of PDMS addition on peel adhesion was analyzed. PIs-3% films had the greatest peeling intensities of 0.98 N/mm and 0.85 N/mm.

## 1. Introduction

Opaque materials are used in conventional electronics [1], hindering light transmission and limiting design options. The demand for innovative and stylish electronic products is rising with technological advancements. Transparent electronic devices are emerging, and open new design possibilities. The market for LED transparent screens is growing, along with challenges [2,3,4]. Polyethylene terephthalate (PET) is used in LED transparent screen materials [5], and is processed through a magnetron sputtering system onto a flexible copper-clad plate. Subsequently, the plate is etched to form a flexible transparent circuit, and lamp beads are embedded through reflow soldering. However, the reflow soldering process requires high temperatures to 288 °C, exceeding the maximum heat resistance temperature (200 °C) of PET. Consequently, low-temperature soldering must be utilized during the embedding process, leading to potential issues such as open welding points and shortened lifespans of the lamp beads. Therefore, developing a transparent flexible copper-clad laminate substrate with high temperature resistance is the key to LED transparent screen research and technological advancement [6,7,8].

The substrate materials for LED transparent screens should have good mechanical properties and high temperature resistance [9,10,11,12], as well as low dielectric constant/loss. A suitable substrate is crucial for circuit board performance and reliability. Common choices for substrates include flexible materials such as polyimide (PI), polytetrafluoroethylene (PTFE), and polyester (PET) film. PET substrates are cost-effective and easily processed, but may degrade in high−temperature settings. PTFE substrates offer excellent dielectric and high-frequency properties, which are suitable for high-frequency and microwave circuits. However, the high hardness and stiffness of PET/PTFE can complicate processing and handling, limiting their flexibility for applications like flexible circuit boards. PI stands out with multiple properties as an exceptional engineering material [13,14,15]. Its remarkable transparency for LED transparent screens can ensure near-complete transparency when the LED screen is inactive, without compromising the background view. Additionally, polyimide boasts excellent thermal stability [16,17,18], which is crucial for the prolonged stable operation of LED screens. The high temperature resistance of PI materials effectively prevents LED component fading or damage during operation. Furthermore, PI exhibits exceptional mechanical strength [19,20,21] and abrasion resistance, safeguarding LED components from external elements and prolonging the lifespan of LED screens. Its compatibility with high−temperature environments and high−frequency applications makes it a promising candidate for flexible circuit boards.

Silicon dioxide (SiO_2_) has been widely used in the field of polyimide doping modification due to its high binding energy and stable structure [22,23,24,25]. However, due to the surface inertness of PI films [26], the interfacial adhesion strength between SiO_2_ and PI film is weak. Moreover, there is a significant difference in the thermal expansion coefficients (CTEs) between SiO_2_ and PI films. As an alternative material, siloxane-modified PI films have attracted increasing attention due to their excellent insulation performance, high and low temperature resistance, and excellent processability [27,28,29,30,31,32]. Siloxane is composed of silicon (Si) and oxygen (O) elements, typically existing as a framework structure of Si-O-Si. This has unique chemical functions, and can interact with polyimide and metal surfaces. The existence of siloxane can improve chemical compatibility between the inherent inorganic metal surface and the organic polyimide matrix. In addition, siloxanes can form chemical bonds and strong interfacial interactions between the PI and metal interface. This behavior includes forming covalent and hydrogen bonds with the PI-metal interface, depending on the properties of the metal and the specific functional groups present in siloxanes.

Polydimethylsiloxane (PDMS) is known for its transparency [33,34,35,36]. It can reduce light absorption and scattering when introduced into PI materials, thereby enhancing transparency and suitability for optical applications. The PI-PDMS layer enhances the adhesion to copper foil, effectively filling microscopic surface irregularities, amplifying the surface contact area [37], and boosting adhesion strength.

Researchers are committed to using no adhesives on the surface of PI-copper foil, which requires increasing intermolecular forces through flexible groups introduction. Sagar M. Doshi prepared PI-copper film with the highest peel adhesion of 930 N/m using concinol tetrarboxylic acid (BTDA) and 4,4′-oxaniline (ODA) [38]. In this study, 1,4-bis(4-amino-2-trifluoromethylphenoxy)benzene (6FAPB) with a trifluoromethyl structure and bis [4-(4-aminophenoxy)phenyl]sulfone (BAPS) with a sulfone structure were chosen as diamine monomers. Bisphenol A-type diethyl ether dianhydride (BPADA) was chosen as a dianhydride monomer. The chosen diamine and dianhydride monomers all feature non-coplanar structures, with flexible structural units like ether bonds, sulfone groups, and trifluoromethyl groups [39,40].

Aminopropyl-capped polydimethylsiloxane (PDMS) was chosen to systematically investigate the interfacial adhesion between PI and copper foil in this study. Its characteristics enhance interfacial adhesion and transparency between synthesized polyimide and copper foil. Polyamide acid (PAA) solutions were prepared by doping with 1%, 3%, and 5% PDMS by weight, and then coating the copper foil. Then, the resulting PI adhesive-free flexible copper−clad laminates were obtained via thermal imidization. The impact of PDMS addition on PI’s thermal stability and mechanical properties were assessed via thermogravimetric and tensile testing. UV spectroscopy testing was employed to evaluate PDMS’s influence on the optical properties of PI. Peel force testing was conducted to analyze the PDMS effect on interfacial adhesion between polyimide and copper foil.

## 2. Materials and Methods

### 2.1. Materials

Bisphenol A diethyl dianhydride (BPADA, 99%) was purchased from Shanghai Tengqian Biotechnology Co., Ltd., Shanghai, China. Aminopropyl-terminated polydimethyloxane (Mw1000), 2,2′-bis (trifluoromethyl)-4.4′-diaminophenyl ether (6FAPB, 99%) and 4,4′-bis (3-aminophenoxy) diphenylsulfone (BAPS, 98%) were both from Shanghai McLean Biochemical Technology Co., Ltd., Shanghai, China. Dimethylacetamide (DMAc) was purchased from China National Pharmaceutical Chemical Reagent Beijing Co., Ltd., Beijing, China.

### 2.2. Synthesis of PDMS-PAA Solution and CPI Copper-Clad Foil

**Scheme 1:**
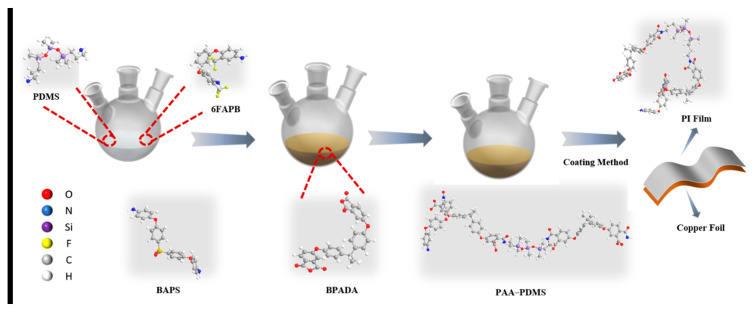
Preparation of CPI copper−clad foil.

The preparation process for the PDMS–PAA solution is shown in Scheme 1. The polymerization experimental formula is shown in Table 1. For example, PI-1/1% colorless transparent polyimide (CPI) film copper-clad sheet was prepared using the following methods. Firstly, 0.049 g (1% molar ratio to diamine) of aminopropyl-terminated polydimethyloxane PDMS and 22.32 g of N,N-dimethylacetamide (DMAc) solvent were added into a 100 mL round bottom flask and stirred for approximately 30 min. After the PDMS completely dissolved, 6.425 g (0.015 mol) of 6FAPB diamine, 7.8072 g (0.015 mol) of BPADA dianhydride, and 10 g of DMAc were added sequentially and mixed continuously at room temperature for 24 h. The copper foil was cleaned with anhydrous ethanol, and then the prepared defoaming PAA solution was evenly spread onto the clean copper foil with a coating machine (MSK-AFA-H200A, Hefei Kejing, Hefei, China) at a speed of 10 mm/s. The copper foil coated with PAA was placed into an oven under nitrogen protection for programmed heating (80 °C, 120 °C, 160 °C, 200 °C, 240 °C, 280 °C, and 300 °C for 20 min each). Polyimide copper-clad foil was obtained after completing the imidization and natural cooling process [41]. The viscosity and Mw of the prepared PAA are shown in Table S1†.

### 2.3. Characterization

The characteristic functional groups of the compounds and polymers were tested using a Nicolet IS10 Fourier infrared spectrometer (Thermo Fisher Scientific, Waltham, MA, USA) in the range of 500–4000 cm^−1^. Wide-angle X-ray diffractions (WA−XRD) were measured on a Rigaku Smart-Lab X-ray diffractometer. Dynamic mechanical (DMA) spectral analysis was performed using a DMA Q800 analyzer (TA, New Castle, DE, USA) under tensile conditions with a 0.01 N preload force, 20 mm amplitude, and 125% force trajectory at a frequency of 1 Hz, as well as a 10 °C/min heating rate. A Pyris 1 TGA thermal analyzer (PerkinElemer, Waltham, MA, USA) was used to investigate the thermal stabilities of the polyimides, with a heating rate of 20 °C min^−1^ and nitrogen flow rate of 20 mL min^−1^ in the range of 30–600 °C. The thermal expansion coefficient (CTE) of PI film was measured using a TMA Q600 analyzer (TA, Delaware, DE, USA), with a temperature range from 50 °C to the top ten degrees of the Tg of PI films, and the heating rate was 10 °C/min. A Lambda−900 UV Visible Spectrometer (Shanghai Perkin Elmer Instrument Co., Ltd., Shanghai, China) was used to test the transmittance of the PI films, with the detection wavelength range between 250 nm and 800 nm. The compressive strength and tensile strength were tested using a universal compressive testing machine (HY0350, Meters Industrial Systems Co., Ltd., Shanghai, China), with a compressive speed of 2 mm/min and a tensile speed of 1 mm/min.

## 3. Results

### 3.1. Chemical Structures

**Figure 1:**
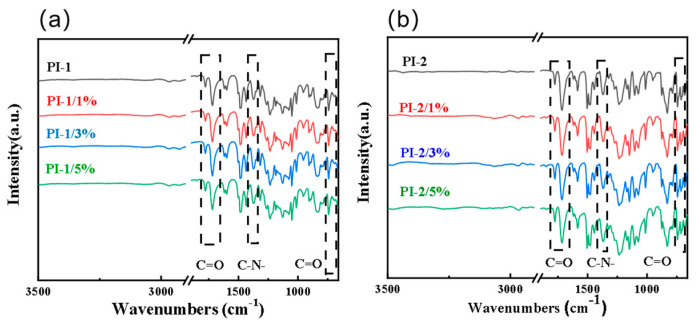
(**a**) FTIR of PIs−1; (**b**) FTIR of PIs−2.

The chemical structures of the PI/PDMS copolymerized films were identified via FTIR, as shown in Figure 1. The characteristic absorption bands at 1720 cm^−1^ and 1780 cm^−1^ can be observed in all curves, which indicate the C=O symmetric and asymmetric vibrations of imide rings. The absorption band at 720 cm^−1^ was caused by the vibration of the imine ring skeleton [42,43,44]. The peak at 1058 cm^−1^ was caused by Si-O-Si skeleton vibration of the PDMS. In addition, the symmetric and asymmetric stretching vibrations of the C-H bonds in the Si-CH_3_ groups at 2960 cm^−1^ and 2900 cm^−1^ are significant characteristic peaks in the PDMS infrared spectra. The bending vibration characteristic peak of the C-H bond in the Si-CH_3_ group is located at 1258 cm^−1^. The C-N bond peaks can be observed at 1380 cm^−1^. The-CF_3_ bands from 6FAPB can be observed at 1296 cm^−1^ [45]. The S=O double bonds from the sulfone groups can be observed from 1250^−1^ to 1350 cm^−1^ at the last two lines.

**Figure 2:**
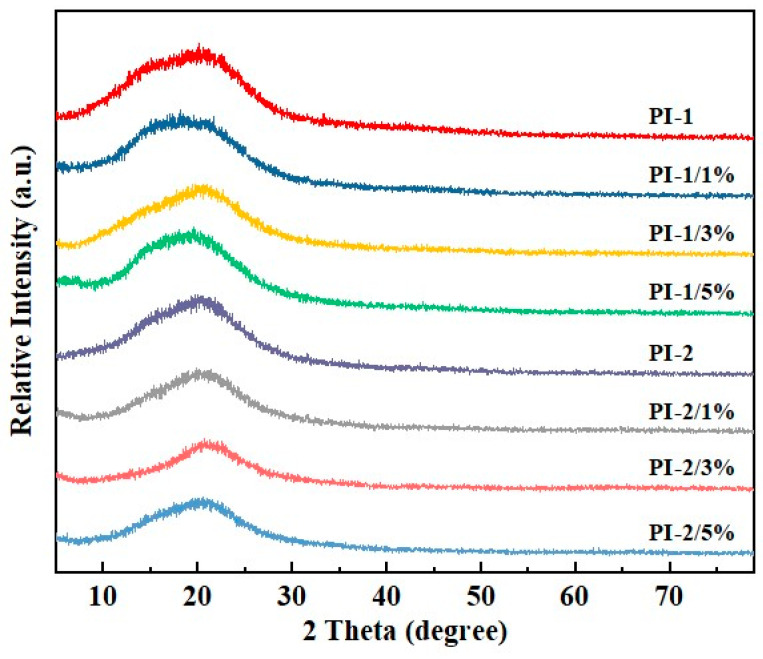
XRD results of polyimide samples with different PDMS contents.

The crystallinities of the prepared polyimide films were studied through XRD, as shown in Figure 2. These X-ray diffraction peaks are mainly concentrated in 2θ = 20°. It can be seen that all of the diffraction curves have non-polar peaks. This indicates that all of the CPI films have an amorphous structure, with amorphous aggregation morphology. Amorphous polyimide films typically have high optical transparency, since they lack ordered crystal structures; this means that the molecular chains are not arranged according to specific rules and directions. When light propagates within PI films, it is not disturbed by regularly arranged molecular chains, thereby reducing light scattering and absorption, resulting in a higher transparency. Amorphous structures can also improve the mechanical properties of PI films. In the amorphous state, the random arrangement of PI molecular chains makes the performance of the films more uniform in all directions, which can effectively disperse external stress and reduce crack propagation. In addition, the tops of all of the XRD peaks are slightly sharp, indicating a certain regular arrangement of the polymer chains [46].

### 3.2. Thermal Properties

**Figure 3:**
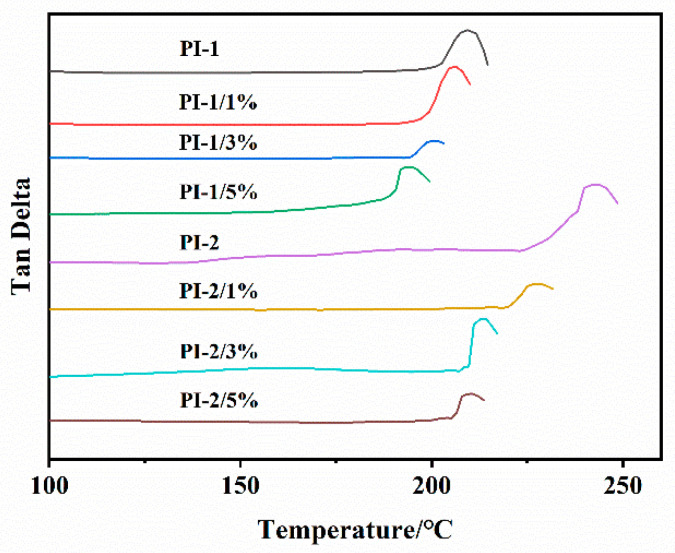
DMA of polyimide samples with different PDMS contents.

The dynamic thermomechanical analyzer was used to test the PDMS-CPI film, and the DMA data are shown in Figure 3. It can be observed that the Tg of PIs-2 was significantly higher than that of PIs-1. The sulfone groups have high polarity in BAPS, and their strong dipole-dipole interactions can significantly enhance intermolecular interactions. This strong intermolecular force not only improves the thermal stability of the PI material, but also allows the polymer chain segments to maintain relatively fixed positions at higher temperatures, thereby increasing the glass transition temperature (Tg) [47]. Although trifluoromethyl is a large and highly electronegative fluorine atom group, its weak overall polarity results in weaker intermolecular forces compared to sulfone groups. This weakens the intermolecular forces of trifluoromethyl containing PI, resulting in a lower glass transition temperature (Tg) [48]. Moreover, the introduction of sulfone groups into the main PI chains can improve the overall rigidity [49]. The shape and size of sulfone groups can promote a more regular chain arrangement and increase the rigidity of PI polymer [50]. The rigid chains are more difficult to move at lower temperatures, thereby increasing the Tg.

With the increase in PDMS, the Tgs of the PIs-1 and PIs-2 films gradually decreased from 231 °C to 217 °C and 266 °C to 232 °C, respectively. This is because the addition of flexible PDMS significantly increased the spatial steric hindrance and flexible silica structure to the PI’s main molecular chains, enhancing the mobility of chain segments. Meanwhile, the PI molecular chains decreased in regularity, and relative slip became easier with the increase in PDMS. The Tg value decreased with the increase in PDMS.

**Figure 4:**
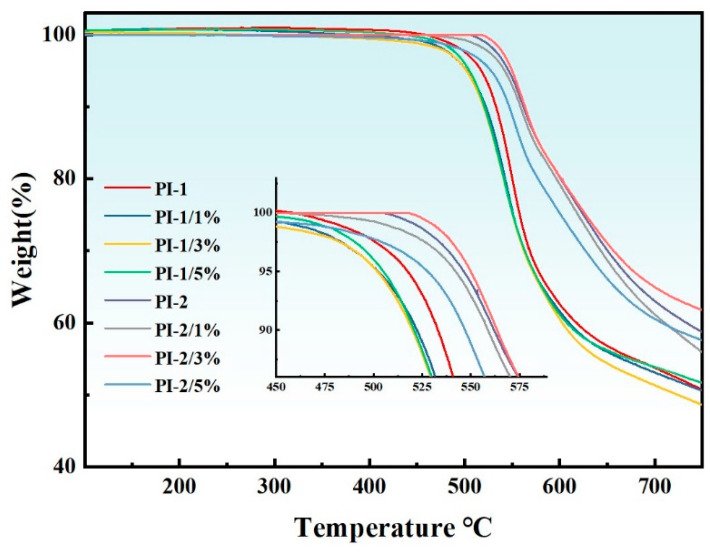
TGA of polyimide samples with different PDMS contents.

**Figure 5:**
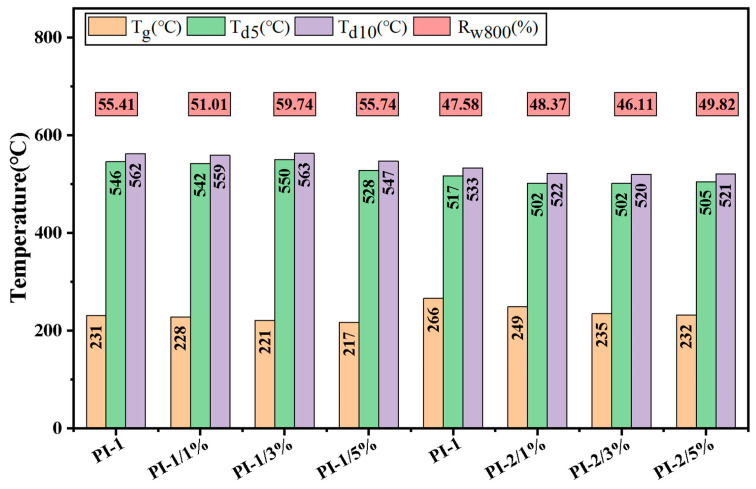
Thermal performances of the prepared PI films. T_d5_: Temperature at 5% weight loss; T_d10_: Temperature at 10% weight loss; Rw800: Remaining mass fraction at 800 °C.

The TGA data are shown in Figure 4, and the related data are shown in Figure 5. The thermal stability of PIs-1 is higher than that of PIs-2. The sulfone group in the BAPS of PI-2 contains sulfur and oxygen elements, which provide high chemical and thermal stability at high temperatures. Sulfur atoms and oxygen atoms can form strong covalent bonds, increasing the rigidity and thermal stability of PI polymer main chains. Sulfone groups, due to their shape and polarity, can promote a more regular and compact arrangement of polymer chains, and sometimes even promote local crystallization [51]. This ordered arrangement and local crystallization can not only improve the mechanical strength of PI material, but also provides better dimensional stability and resistance to thermal deformation at high temperatures. The regular molecular arrangement and local crystallization zone can effectively block the molecular chain slip and fracture caused by thermal energy. The thermal decomposition temperature of the polymer is at its highest, beyond which the polymer begins to decompose. The combined effect of sulfone’s chemical structure and its enhancement of intermolecular forces increased the thermal decomposition temperature of the PI polymers. Sulfone groups containing polyimides can remain stable at higher temperatures. In contrast, trifluoromethyl-containing polyimides cannot significantly improve thermal stability compared to sulfone groups. The larger trifluoromethyl group may lead to looser arrangements of PI polymer chains, resulting in poorer performance in high−temperature applications compared to sulfone-based PI.

With the continuous increase in PDMS, the thermal weight loss temperatures of T_d5%_ and T_d10%_ both decreased, from 546 °C to 528 °C and from 517 °C to 505 °C, respectively. The RW800 temperatures of PIs-1 are all above 51%, and the RW800 values of PIs-2 are all above 46%. This is because a small amount of PDMS had not taken part in the polymerization and was independent in the polyimide molecular chains. Another reason is that the reaction activity of the diamines was different, which reduced the degree of polymerization and led to a decrease in thermal stability.

**Figure 6:**
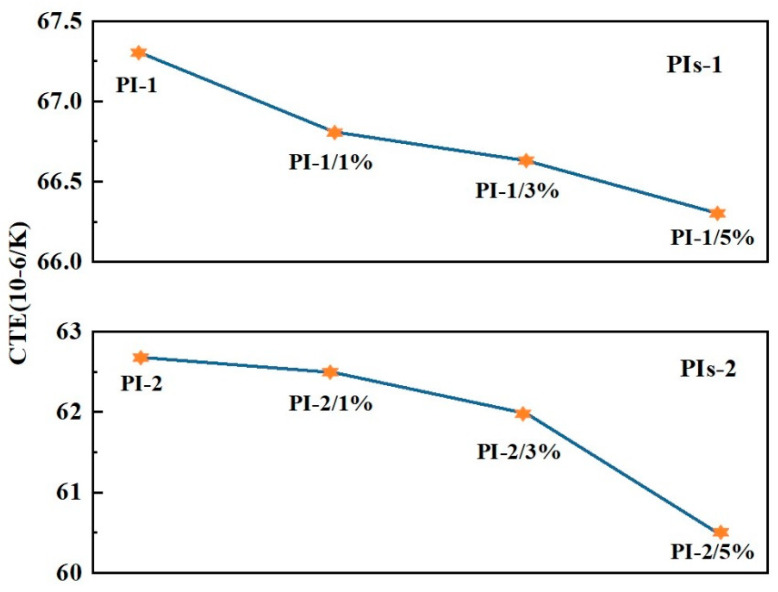
Thermal expansion coefficients of polyimides with different contents of PDMS.

Figure 6 shows the coefficients of thermal expansion (CTE) of the CPI/PDMS copolymerized films. It can be observed that the CTE of PIs-2 film is lower than that of PIs-1 film. The sulfone groups contained in PIs-2 increased the polar interactions between PI polymer chains (such as hydrogen bonds). These interactions can significantly enhance the intermolecular forces and increase the overall molecular rigidity. Trifluoromethyl groups in PIs-1 have polarity and high electronegativity, which can increase the interaction forces between PI polymer molecules and reduce the thermal expansion of PI material. However, in comparison, the polarity and interaction forces of sulfone groups are more effective in reducing the CTE than trifluoromethyl groups.

The CTE of PI decreased accordingly as PDMS increased. This is due to the flexibility and chain mobility of PDMS, which can introduce additional intermolecular space in the PI material and reduce intermolecular interactions. This effect facilitated sliding between PI molecules when heated, and reduced the CTE values.

### 3.3. Optical Properties

**Figure 7:**
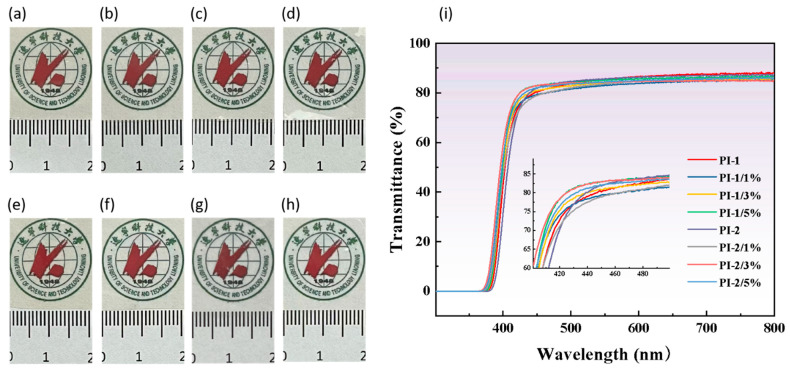
(**a**–**h**) Photographs of PI-1, PI-1/1%, PI-1/3%, PI-1/5%, PI-2, PI-2/1%, PI-2/3%, and PI-2/5%; (**i**) UV–visible spectra of polyimide films with different contents of PDMS.

According to Figure 7, it can be seen that the color and optical properties of the PIs-1 and PIs-2 series are not significantly different. All of the PI films have high transparency because the sulfone and trifluoromethyl groups do not absorb light in the visible light region. The optical performance of the PIs-1 film is superior to that of the PIs-2 film. The introduction of trifluoromethyl groups in PIs-1 films made the molecular chains stack more loosely, which reduced light scattering in the PI material and increased its light transmittance. The polarity of the sulfone groups in PIs-2 is stronger than that of trifluoromethyl, which makes the molecular chains more inclined towards a tight arrangement, leading to increased light scattering. The UV-visible data are shown in Table S2†. The thickness data of the prepared films are shown in Table S3†. As the content of PDMS increased, the color and transmittance showed a trend of first decreasing, and then increasing. This is because PDMS doping can introduce interface effects, resulting in an uneven interface between PDMS and PI, and leading to optical scattering. Doping with a small amount of PDMS introduced voids or defects into the PI micro-structure, which can lead to multiple scattering of light, reducing its transmittance. Doping with a large amount of PDMS can inhibit the formation of a CTC (charge transfer complex effect), and forms an anti-reflective effect, reducing the reflectivity of the PI surface. The PI-1/3%, PI-1/5%, PI-2/3%, and PI-2/5% films have higher transmittances exceeding 80% at 450 nm. This indicates that after the addition of PDMS, the PI film can still maintain high transparency. The result of this research has reference significance to the development of high-performance transparent PI films, especially in applications that require high transmittance, such as optoelectronic devices, flexible displays, transparent conductive films, and so on.

### 3.4. Mechanical Properties

**Figure 8:**
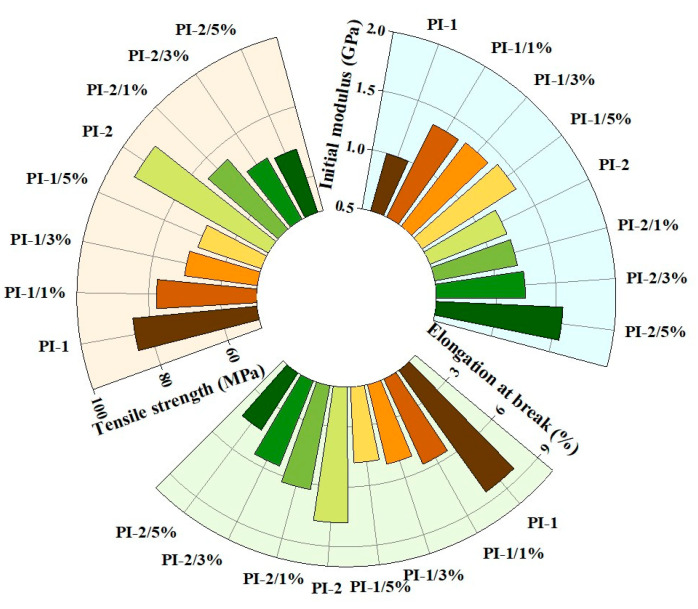
Mechanical properties of polyimide samples with different contents of PDMS.

As a high-performance engineering plastic, the mechanical properties of PI films are crucial for many applications. Figure 8 shows that the mechanical properties of PIs-2 are higher than those of PIs-1. This is because BAPS introduces sulfone groups, increasing the rigidity of polyimide molecular chains. The rigid chain segment reduces the deformation of PI material under external forces, and improves the elastic modulus and bending resistance. Rigid molecular chains also mean PI materials can better maintain their shape, and are less prone to deformation when subjected to external forces. The polarity and shape of sulfone groups can promote a more regular and dense arrangement of PI polymer chains, and can also promote local crystallization. This improvement in crystallinity can significantly enhance the hardness and modulus of PI materials. The crystalline region is ordered at the molecular level, and can effectively disperse and bear more mechanical stress [52]. In 6FAPB, the spatial steric hindrance of fluorine atoms weakens intermolecular forces, resulting in lower interchain bonding strength [42]. The elongation at break, tensile strength, and elastic modulus gradually decreased as the PDMS content increased. The different reactivities between PDMS and diamine monomers caused decreased degrees of polymerization during monomer copolymerization, resulting in molecular weight decreases and decreased mechanical properties. In addition, PDMS introduced flexible groups and twisted non-coplanar structures, resulting in decreased polymer chain regularity. The PI films became prone to fracture under mechanical tension.

### 3.5. Adhesion Testing and Peel Adhesion

The HY0350 electronic universal machine from Meters Industrial Systems Co., Ltd., Tokyo, Japan, was used to test the bonding force between the PI film and copper foil by clamping and stretching the PI film end and the copper foil end, respectively [53]. The sample size is 20 cm × 20 mm. The peel strength was calculated according to Formula (1):

σ180° = F/B (1)

where

σ180°—Peel strength, N/mm;

F—Peeling force, N;

B—Sample width, mm.

**Figure 9:**
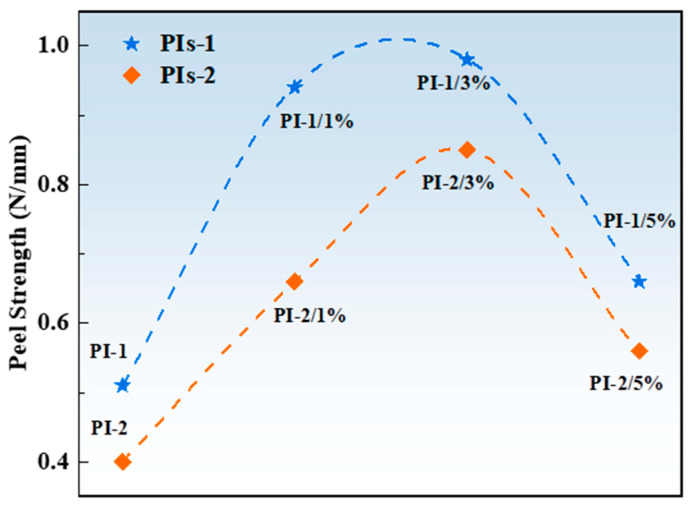
Peel strengths of polyimide samples with different contents of PDMS.

The 180° peel adhesion data are displayed in Figure 9. The peel adhesion values of PI-1 and PI-2 are 0.51 N/mm and 0.44 N/mm, respectively. This indicates that oxygen (sulfone group) may bind with silicon in the PI-2 series, leading to chemical bonds decreasing and a lower peel adhesion with copper foil. All of the copper−coated films exhibited an increase in peel adhesion as the PDMS content increased. The adhesion strength between PI–copper foil films without PDMS doping was the worst, while the adhesion strength between PI–copper foil films increased as PDMS doping increased. PI-1/3% and PI-2/3% have the highest peel adhesions, with peel adhesions of 0.98 N/mm and 0.85 N/mm, respectively. This is due to PDMS having higher surface energy and adhesion energy, which can lead to stronger adhesion with copper foil and improvement in the adhesion strength. The functional groups in PDMS molecules may also form hydrogen or covalent bonds with the oxide layer on the copper foil surface, enhancing the interaction force between PI and copper foil [54]. The introduction of PDMS can reduce the interface energy difference between PI and copper foil [55]. In addition, the flexibility and toughness of PDMS molecules can disperse external stress, and slow down the crack propagation speed [56]. This prevented cracks expanding from the interface, and delayed the peeling process of the PI-copper foil. The peel adhesion decreased when the PDMS doping was 5%. This proves that excessive addition of PDMS can also lead to a decrease in interfacial bonding strength. The increase in PDMS leads to a decrease in the polymerization degree of polymer chains. PDMS macromolecules exist in free form in PAA solution. Secondly, excessive PDMS may hinder the effective bonding between the copper-coated film and PI substrate, leading to a decrease in peel adhesion.

## 4. Conclusions

Two diamine monomers (6FAPB, BAPS) and dianhydride monomer (BPADA) were selected to prepare copper−coated PI films using a coating method and chemical imidization method. The influences of sulfone and trifluoromethyl groups of polyimide-copper-coated films on the mechanical properties, thermal properties, and optical properties were systematically analyzed.

PDMS was doped at 0% wt, 1% wt, 3% wt and 5% wt in each series. The effects of PDMS on interfacial adhesion between PI-copper were analyzed via 180° peel adhesion experiments. The PDMS 3% wt series had the greatest peel adhesion, with 0.98 N/mm for PI-1/3% and 0.85 N/mm for PI-2/3%. This is because the interaction forces between PDMS molecule groups and the oxide layer of the copper-clad substrate, which improves the binding strength between the PI and copper foil. The PDMS 5% wt films were more in free form in PAA solution, which hindered the effective binding between the copper-coated film and the substrate. Compared with the study of Jin et al. [53], they designed and fabricated a high-power HWP device for PI film surface treatment. After plasma treatment and copper plating, the PI film had good adhesion to the Cu film (peel strength > 0.8 N/mm).

The DMA and TGA results showed that the T_d 5%_, T_d 10%_ heat loss temperature, and Tg decreased gradually as PDMS doping increased. The ultraviolet spectral test showed that as the PDMS content increased, the transmittance of PI film decreased first, and then increased. All of the PI films have high transparency because the sulfone and trifluoromethyl groups do not absorb light in the visible light region. The optical performance of the PIs-1 film is superior than that of the PIs-2 film. As the content of PDMS increased, the color and transmittance showed a trend of first decreasing, and then increasing. Doping with a large amount of PDMS can inhibit the formation of a CTC (charge transfer complex effect), and forms an anti−reflective effect, reducing the reflectivity of the PI surface. The PI-1/3%, PI-1/5%, PI-2/3%, and PI-2/5% films have higher transmittances, exceeding 80% at 450 nm. This is because doping with small amounts of PDMS introduced voids, and caused light scattering multiple times in the material, reducing the transmittance of light. In this research, the resulting optical performance and stripping strength meet the requirements of flexible copper-clad substrates. The prepared PI-PDMS films are expected to be used in transparent LED screen applications.

## Figures and Tables

**Table 1:** Experimental formulations of 6FAPB/BAPS−BPADA polyimide.

System Name	n_(6FAPB)_^a^ (mmol)	n_(BAPS)_^a^ (mmol)	n_(BPADA)_^a^ (mmol)	Mass Fraction of PDMS (%) ^b^	Remark
PI-1	15	/	15	/	film
PI-1/1%	15	/	15	1	film
PI-1/3%	15	/	15	3	film
PI-1/5%	15	/	15	5	film
PI-2	/	15	15	/	film
PI-2/1%	/	15	15	1	film
PI-2/3%	/	15	15	3	film
PI-2/5%	/	15	15	5	film

^a^ Molar amount of 6FAPB, BAPS, BPADA in PI film; ^b^ Mass percentage of PDMS added in PI film.

## Data Availability

Data are contained within the article.

## Supplementary Materials

The following supporting information can be downloaded at: https://www.mdpi.com/article/10.3390/polym16111591/s1, Table S1: Mw and Viscosity of the prepared PI films; Table S2: UV−visible data of the prepared PI films; Table S3: The thickness of the prepared PI films.
