# Supplementary material for: Adhesion and Transparency Enhancement between Flexible Polyimide-PDMS Copolymerized Film and Copper Foil for LED Transparent Screen
Source: Polymers (Basel). 2024 Jun 4;16(11):1591. doi: 10.3390/polym16111591 (PMC11174799; doi:10.3390/polym16111591)
Supplement: Supplementary file 1 [file polymers-16-01591-s001.zip › polymers-3032686-supplementary.pdf]

Adhesion and Transparency Enhancement between Flexible Polyimide–PDMS Copolymerized Film and Copper Foil for LED Transparent Screen

Xinming Wang [a], Yuting Zhao [a], Heming Li [a], Weiguo Gao [a],Yan Liu [a], Anning Sun [a], Ke Ma [a,*], Zhizhi Hu [a,b] * and Yongqi Wang [c,*]

[a]School of Chemical Engineering, University of Science and Technology Liaoning, Anshan 114051, China
[b]School of Chips, XJTLU Entrepreneur College (Taicang), Xi'an Jiaotong–Liverpool University, Taicang, Suzhou 215400, China
[c]School of Information and Engineering, Liaoning Agricultural Technical College, Yingkou, Liaoning, PR China
*Correspondence: mkustl@163.com (K.M.); zzhustl@163.com (Z.H.); wyq_sj214@163.com (Y.W.)

Appendix

Table S1†. Mw and Viscosity of the prepared PI films.

Samples	M_w	Viscosity，MPa·s
PI–1	219571	24000
PI–1/1%	188403	10000
PI–1/3%	188530	7300
PI–1/5%	174798	5400
PI–2	188670	18300
PI–2/1%	116372	8200
PI–2/3%	115824	5400
PI–2/5%	82866	3600

Table S2†. UV–visible data of the prepared PI films.

Samples	Transmittance(%) at 450nm wavelength
PI–1	80.22%
PI–1/1%	79.40%
PI–1/3%	80.82%

PI−1/5%	82.98%
PI−2	81.80%
PI−2/1%	78.93%
PI−2/3%	81.98%
PI−2/5%	82.90%

Table S3†. The thickness of the prepared PI films.

Samples	Thickness (mm)
PI−1	0.051
PI−1/PDMS−1%	0.021
PI−1/PDMS−3%	0.028
PI−1/PDMS−5%	0.024
PI−2	0.045
PI−2/PDMS−1%	0.03
PI−2/PDMS−3%	0.05
PI−2/PDMS−5%	0.036
PI−3	0.064
PI−3/PDMS−1%	0.03
PI−3/PDMS−3%	0.037
PI−3/PDMS−5%	0.028
